# Sleeplessness in Infants

**Published:** 1870-10

**Authors:** 


### SLEEPLESSNESS IN INFANTS.

By far the most common cause of restlessness at night is *injudicious feeding*, the child being stuffed with food, which, although not necessarily in itself injurious, is yet ill-adapted to the nourishment of the particular infant to whom it is given. It is a common practice among mothers—especially those of the poorer classes—to make up for any deficiency in the amount of breast-milk by farinaceous food, long before the digestive power of the child is suited to such a diet. The stomach of an infant of about two months old is thus often filled with a mass of starchy matters, which the absence of saliva will not permit him to digest. This mass, fermenting in his bowels, is a source of continual discomfort until it is evacuated. Even when cow's milk is used as an addition to breast-milk, it is frequently ill-digested, although diluted with water. The clot formed by the coagulation of cow's milk is particularly firm and solid and differs very much in that respect from the clot of human milk, which is exceedingly light and flocculent. In very young infants, therefore, and in older infants of delicate stomachs, the digestive juices can make little impression upon the mass of curd. Feeding so conducted cannot be continued for long together, without producing very evident signs that the nutrition of the body is no longer efficiently maintained. The child, deriving very little nourishment from the food, which, however, he eagerly swallows, will soon begin to waste, in spite of his voracity. But, before nutrition has become impaired so decidedly as to produce emaciation, certain symptoms are noticed, showing the uneasiness of the digestive organs; and one of the earliest of these signs is restlessness at night.—The child starts out of his sleep, crying violently. His skin is hot, his belly tense, his upper lip livid and drawn up at the corners, and the griping pains from which he is suffering are shown by the violent contortions of his body, and the uneasy, jerking movements of the limbs. Even when taken up into the arms of his mother he is not pacified, but breaks out into piercing cries, which nothing is able to quiet until he becomes exhausted. Other signs of his unsuitable food consist in frequent hiccough, flatulence, sour eructations, and constipations.—The sluggishness of the bowels is due to excessive secretion of mucus in the alimentary canal, excited by constantly renewed irritation of its lining membrane. The mucous being coagulated by the acid, resulting from the decomposition of starchy matters, covers the masses of food, and lines the inner surface of the bowel, so that the slippery surfaces glide over one another, and the contents of the intestine are not forced along. The stools themselves consist of little round masses, remarkably firm, and of a yellowish color, exhibiting, when crushed, a cheesy appearance. They are evidently composed of curds and undigested farinaceous matter. The smell is often offensively sour, and they are accompanied by a considerable quantity of tough mucous, either covering the little lumps, or appearing in the form of strings, which have been mistaken for portions of parasitic worms.

The cause of wakefulness at night is so excessively common, that in every case where this distressing symptom is complained of, inquiry should at once be made into the diet of the infant, so that, by a proper adjustment of the quality and quantity of his powers of digestion, the child may be supplied with a diet which he is able completely to assimilate. When this has been done, and the bowels have been assisted by a gentle laxative to expel the undigested contents, the improvement is immediate; the child sleeps soundly, and his irritability ceases at once.

It must be remembered that plumpness in an infant is no proof that his feeding is judiciously conducted. Badly fed children may be exceedingly fat, as we sometimes see in cases of commencing rickets, where the adipose tissue is in great excess, although the general nutrition of the body is by no means satisfactory.—*British Medical Journal.*

---

GOOD NEWS, IF TRUE.—It is stated that M. Tessiè du Montay (may his shadow never be less!) has devised an oxyhydrogen light, in which no pencil of lime, magnesia, or zircon is required. Supercarburetted hydrogen is used instead of ordinary hydrogen. The new light is wonderfully cheap, as well as brilliant, costing only two centimes (*less than half a cent*) for five hours. If there is no mistake about this (and it comes on good authority), the old-fashioned mode of gas-lighting may be considered as "played out."

---

COLOGNE VINEGAR.—To one pint of good *eau de cologne*, add halt an ounce of strong acetic acid. This mixture is much used in France as n application in nervous headaches, etc.

